# Brain-Derived Neurotrophic Factor Suppressed Proinflammatory Cytokines Secretion and Enhanced MicroRNA(miR)-3168 Expression in Macrophages

**DOI:** 10.3390/ijms23010570

**Published:** 2022-01-05

**Authors:** Hui-Chun Yu, Hsien-Bin Huang, Hsien-Yu Huang Tseng, Ming-Chi Lu

**Affiliations:** 1 Division of Allergy, Immunology and Rheumatology, Dalin Tzu Chi Hospital, Buddhist Tzu Chi Medical Foundation, Chiayi 62247, Taiwan; df928039@tzuchi.com.tw (H.-C.Y.); david_hthy@hotmail.com (H.-Y.H.T.); 2 Department of Life Science and Institute of Molecular Biology, National Chung Cheng University, Minxiong 621301, Taiwan; biohbh@ccu.edu.tw; 3 School of Medicine, Tzu Chi University, Hualien City 97004, Taiwan

**Keywords:** BDNF, p75NTR, JNK, macrophages, proinflammatory cytokines, microRNAs

## Abstract

We investigated the role of brain-derived neurotrophic factor (BDNF) and its signaling pathway in the proinflammatory cytokines production of macrophages. The effects of different concentrations of BDNF on proinflammatory cytokines expression and secretion in U937 cell-differentiated macrophages, and human monocyte-derived macrophages were analyzed using enzyme-linked immunosorbent assay and real-time polymerase chain reaction. The CRISPR-Cas9 system was used to knockout p75 neurotrophin receptor (p75NTR), one of the BDNF receptors. Next-generation sequencing (NGS) was conducted to search for BDNF-regulated microRNA. A very low concentration of BDNF (1 ng/mL) could suppress the secretion of interleukin (IL)-1β, tumor necrosis factor (TNF)-α, and IL-6 in lipopolysaccharide (LPS)-stimulated macrophages but did not change their mRNA expression. BDNF suppressed IL-1β and IL-6 secretion in human monocyte-derived macrophages. In U937 cells, BDNF suppressed the phosphorylation of JNK and c-Jun. The p75NTR knockout strongly suppressed IL-1β, IL-6, and TNF-α secretion in macrophages and LPS-stimulated macrophages. BDNF regulated the expression of miR-3168 with Ras-related protein Rab-11A as its target. In conclusion, BDNF suppressed proinflammatory cytokines secretion in macrophages and inhibited the phosphorylation of JNK. Knockout of p75NTR suppressed proinflammatory cytokines expression and secretion. BDNF upregulated the expression of miR-3168. The inhibition of p75NTR could be a potential strategy to control inflammation.

## 1. Introduction

Brain-derived neurotrophic factor (BDNF) is well known for its role in the differentiation, maturation, and survival of neurons. BDNF is involved in the pathogenesis of many neuropsychiatric diseases [1,2] and serum levels of BDNF are altered in many neuropsychiatric diseases, such as depression [3]. Inflammatory responses also contribute to the pathogenesis of these neuropsychiatric diseases [4]. Elevated serum levels of BDNF were found in some autoimmune diseases, such as Sjögren’s syndrome, systemic lupus erythematosus, and rheumatoid arthritis, and altered serum levels of BDNF was associated with disease activity or medication used in patients with these diseases [5,6,7,8]. As these reports suggest that BDNF might be directly participating in the inflammatory response, human immune cells could produce BDNF [9]. Recently, BDNF was suggested to play a role in the pathogenesis of rheumatoid arthritis [10].

Our previous study showed that BDNF could increase *interleukin* (*IL*)*-2*, *IL-17*, and *interferon* (*IFN*)*-γ* expression in Jurkat cells [7]. Macrophages are not only the first-line responders to microbial infections, but also play critical roles in the immunopathogenesis of many diseases [11]. We noted that two of the BDNF receptor p75 neurotrophin receptor (p75NTR) and tropomyosin receptor kinase B (TrkB) were expressed on macrophages and these two receptors could affect the differentiation and survival of macrophages [12,13,14]. However, the effects of BDNF and its signaling pathway on the proinflammatory cytokine secretion in macrophages are still unclear. Therefore, we hypothesized that BDNF could affect the secretion of proinflammatory cytokines in macrophages. In this study, we studied the effects of BDNF on tumor necrosis factor (TNF)-α, IL-1β, and IL-6 secretion in U937 cell-differentiated macrophages and human monocyte-derived macrophages. We also investigated the related signaling pathways and the effects of BDNF on the expression of miRNAs and its potential targets.

## 2. Results

### 2.1. Effects of BDNF on IL-1β, IL-6, and TNF-α Secretion and mRNA Expression in U937 Cells, Differentiated Macrophages, and Lipopolysaccharide (LPS)-Stimulated Macrophages

**Figure 1:**
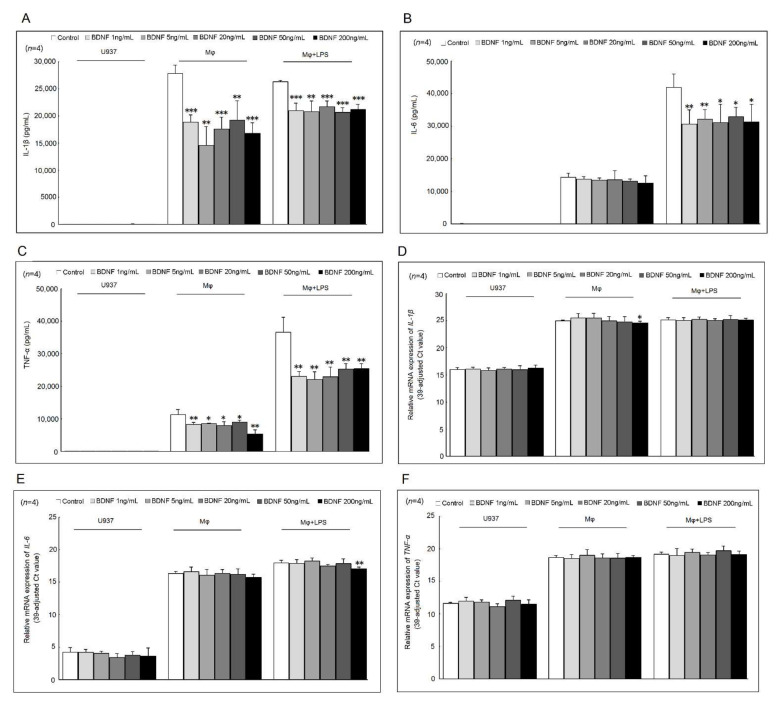
Effects of BDNF on IL-1β, IL-6, and TNF-α secretion and mRNA expression. U937 cells, differentiated macrophages, and LPS-stimulated macrophages were cultured with different concentrations of BDNF (0, 1, 5, 20, 50, and 200 ng/mL) for 48 h (for cytokine secretion) or 4 h (for mRNA expression). The effects of different concentrations of BDNF for the secretion of (**A**) IL-1β, (**B**) IL-6, and (**C**) TNF-α in U937 cells, differentiated macrophages, and LPS-stimulated macrophages. The effects of different concentrations of BDNF for the mRNA expression of (**D**) *IL-1β*, (**E**) *IL-6*, and (**F**) *TNF-α* in U937 cells, differentiated macrophages, and LPS-stimulated macrophages (* *p* < 0.05; ** *p* < 0.01; *** *p* < 0.001).

The addition of different concentrations (1, 5, 20, 50, and 200 ng/mL) of BDNF could suppress IL-1β and TNF-α, but not IL-6 secretion in differentiated macrophages compared with those cultured with medium only (Figure 1A–C). In LPS-stimulated macrophages, the cytokine secretion of IL-1β, TNF-α, and IL-6 were all significantly decreased after cultured with different concentrations (1, 5, 20, 50, and 200 ng/mL) of BDNF (Figure 1A–C). As expected, the mRNA expression of *IL-1β*, *TNF-α*, and *IL-6* was increased in differentiated macrophages or LPS-stimulated macrophages compared with U937 cells. Only the addition of a high concentration of BDNF (200 ng/mL) could suppress the mRNA expression levels of *IL-1β* in differentiated macrophages and those of *IL-6* in LPS-stimulated macrophages compared with those cultured with medium only (Figure 1D–F).

### 2.2. Effects of BDNF in Mitogen-Activated Protein Kinase (MAPKs) and c-Jun Phosphorylation

Next, we investigated the effect of BDNF on the downstream MAPKs and c-Jun phosphorylation. We analyzed the effect of different concentrations of BDNF on MAPKs and c-Jun phosphorylation in U937 cells.

**Figure 2:**
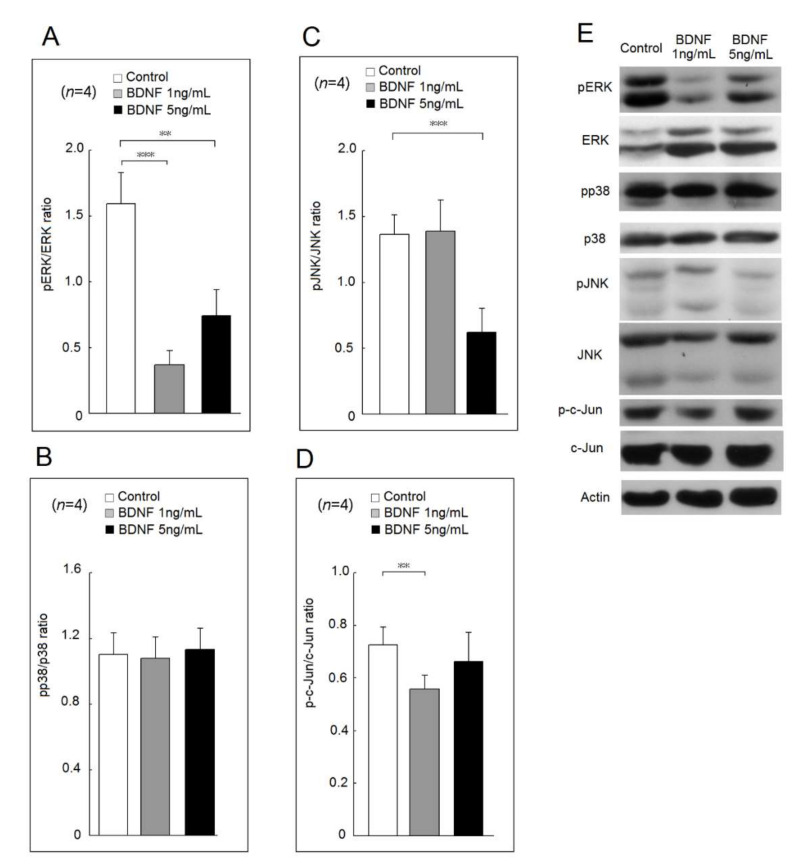
Effects of BDNF at concentrations of 1 ng/mL and 5 ng/mL in mitogen-activated protein kinase (MAPK) and c-Jun phosphorylation of U937 cells. The phosphorylation ratio of (**A**) extracellular signal-regulated kinases (ERK), (**B**) p38, (**C**) c-Jun N-terminal kinases (JNK) and (**D**) c-Jun in U937 cells after cultured with medium only, low concentration of BDNF (20 ng/mL) or high concentration of BDNF (200 ng/mL) for 48 h. (**E**) A representative case (** *p* < 0.01; *** *p* < 0.001).

In Figure 2, both BDNF at 1 ng/mL or 5 ng/mL suppressed the phosphorylation of extracellular signal-regulated kinases (ERK) compared with culture medium only (Figure 2A). BDNF at 1 ng/mL or 5 ng/mL did not affect the phosphorylation ratio of p38 (Figure 2B). In Figure 2C, BDNF at 5 ng/mL decreased the phosphorylation of c-Jun N-terminal kinases (JNK). BDNF at 1 ng/mL but not 5 ng/mL suppressed the phosphorylation of c-Jun (Figure 2D). A representative example is shown in Figure 2E.

**Figure 3:**
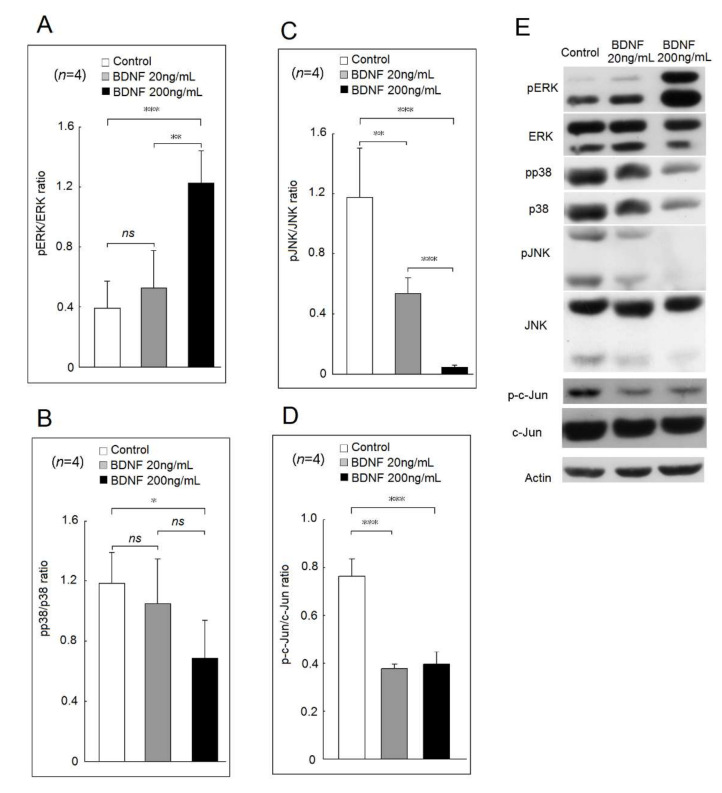
Effects of BDNF at concentrations of 20 ng/mL and 200 ng/mL in mitogen-activated protein kinase (MAPK) and c-Jun phosphorylation of U937 cells. The phosphorylation ratio of (**A**) extracellular signal-regulated kinases (ERK), (**B**) p38, (**C**) c-Jun N-terminal kinases (JNK) and (**D**) c-Jun in U937 cells after cultured with medium only, low concentration of BDNF (20 ng/mL) or high concentration of BDNF (200 ng/mL) for 48 h. (**E**) A representative case (ns, not significant; * *p* < 0.05; ** *p* < 0.01; *** *p* < 0.001).

In Figure 3, a high concentration of BDNF (200 ng/mL) could increase the phosphorylation ratio of ERK compared with BDNF at 20 ng/mL or the culture medium only (Figure 3A). A high concentration of BDNF (200 ng/mL) could suppress the phosphorylation of p38 compared with the culture medium only (Figure 3B). Finally, both BDNF at 20 ng/mL or 200 ng/mL could suppress the phosphorylation of JNK and c-Jun (Figure 3C,D). A representative example is shown in Figure 3E.

### 2.3. Effects of BDNF on Proinflammatory Cytokine Secretion in Human Monocyte-Derived Macrophages

**Figure 4:**
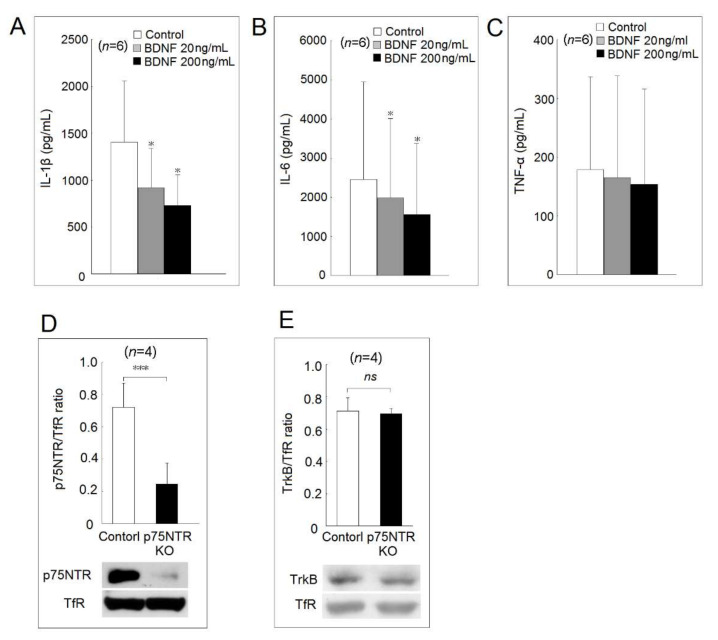
Effects of different concentrations of BDNF on (**A**) IL-1β, (**B**) IL-6, and (**C**) TNF-α secretion in human monocyte-derived macrophages. U937 cells were transfected with CRISPR-Cas9 plasmid containing gRNA that targets p75NTR or control CRISPR-Cas9 plasmid as the control group. The cell surface expression (**D**) p75NTR and (**E**) TrkB on U937 cells were analyzed by Western blot analysis (ns, not significant; * *p* < 0.05; *** *p* < 0.001).

The effects of BDNF on human monocyte-derived macrophages were further explored. Either a low or a high concentration of BDNF could suppress IL-1β and IL-6, but not TNF-α secretion in human monocyte-derived macrophages (Figure 4A–C).

### 2.4. The Expression of Two BDNF Receptors, TrkB and p75NTR Expression in p75NTR Knockout U937 Cells

We analyzed the protein expression of the two BDNF receptors, TrkB and p75NTR, in U937 cells after p75NTR knockout using the CRISPR-Cas9 system. We confirmed that the membrane protein expression of p75NTR, but not TrkB, was dramatically decreased after p75NTR knockout (Figure 4D,E).

### 2.5. Effect of BDNF on U937 Cell Viability and Proliferation

**Figure 5:**
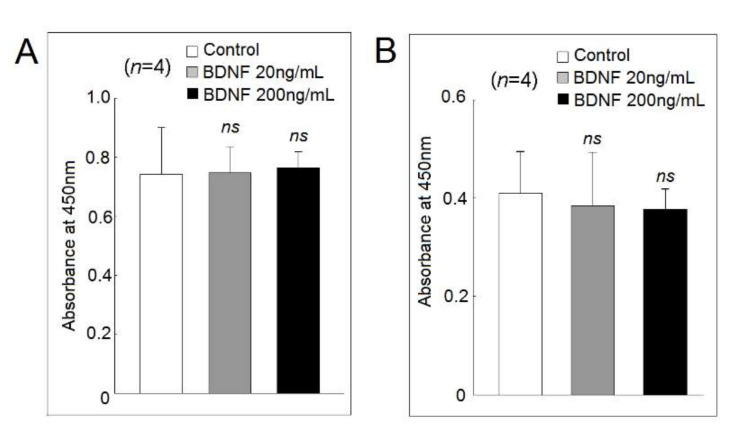
Effects of different concentrations of BDNF on (**A**) U937 cells and (**B**) differentiated macrophages viability and proliferation analyzed using the mitochondrial dehydrogenase cleavage assay (ns, not significant).

We found that both low and high concentrations of BDNF did not affect the cell viability and proliferation of U937 cells (Figure 5A) or differentiated macrophages (Figure 5B).

### 2.6. Effects of p75NTR Knockout on IL-1β, IL-6, and TNF-α Secretion and mRNA Expression

**Figure 6:**
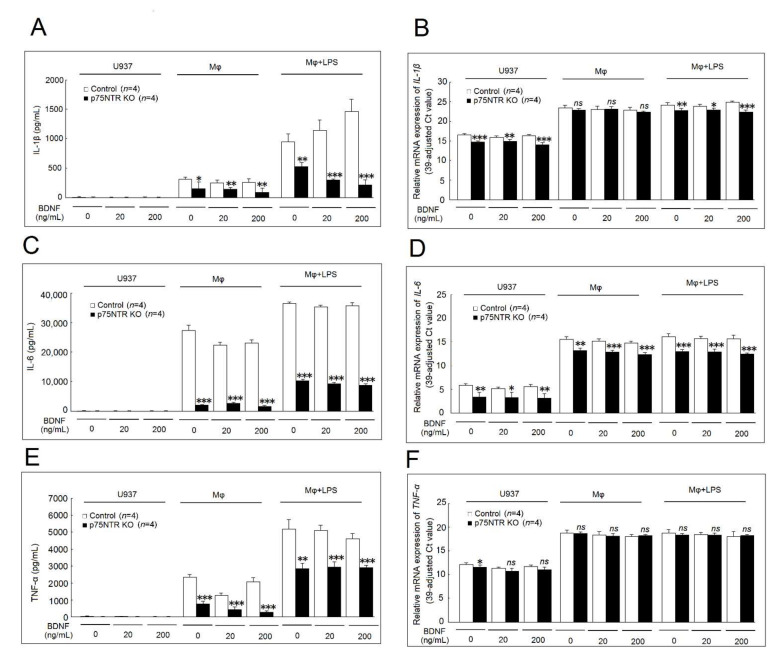
Effects of p75NTR knockout on IL-1β, IL-6, and TNF-α secretion and mRNA expression. Controls or p75NTR knockout U937 cells were further induced to differentiate into macrophages and then stimulated with LPS with the presence of culture medium, a low concentration of BDNF (20 ng/mL), or a high concentration of BDNF (200 ng/mL) for 48 h (for cytokines secretion) or 4 h (for mRNA expression). The effects of p75NTR knockout on IL-1β (**A**) secretion and (**B**) mRNA expression. The effects of p75NTR knockout on IL-6 (**C**) secretion and (**D**) mRNA expression. The effects of p75NTR knockout on TNF-α (**E**) secretion and (**F**) mRNA expression (ns, not significant; * *p* < 0.05; ** *p* < 0.01; *** *p* < 0.001).

We found that p75NTR knockout could effectively suppress IL-1β secretion in differentiated macrophages and LPS-stimulated macrophages cultured in medium, at low and high concentrations of BDNF compared with the controls (Figure 6A). The mRNA expression of *IL-1β* was significantly decreased in p75NTR knockout U937 cells and LPS-stimulated macrophages, but not in differentiated macrophages cultured with medium, at low and high concentrations of BDNF compared with controls (Figure 6B). Figure 6C shows that p75NTR knockout effectively suppressed IL-6 secretion in differentiated macrophages and LPS-stimulated macrophages cultured with medium, at low and high concentrations of BDNF compared with the controls. The mRNA expression of *IL-6* was significantly decreased in p75NTR knockout U937 cells, differentiated macrophages, or LPS-stimulated macrophages cultured with medium, at low and high concentrations of BDNF compared with the controls (Figure 6D). Figure 6E shows that p75NTR knockout also effectively suppressed TNF-α secretion in differentiated macrophages and LPS-stimulated macrophages cultured with medium, at low and high concentrations of BDNF compared with the controls. The p75NTR knockout only slightly suppressed the mRNA expression of *TNF-α* in U937 cultured with medium only (Figure 6F).

### 2.7. Expression of miRNAs Regulated by BDNF

**Figure 7:**
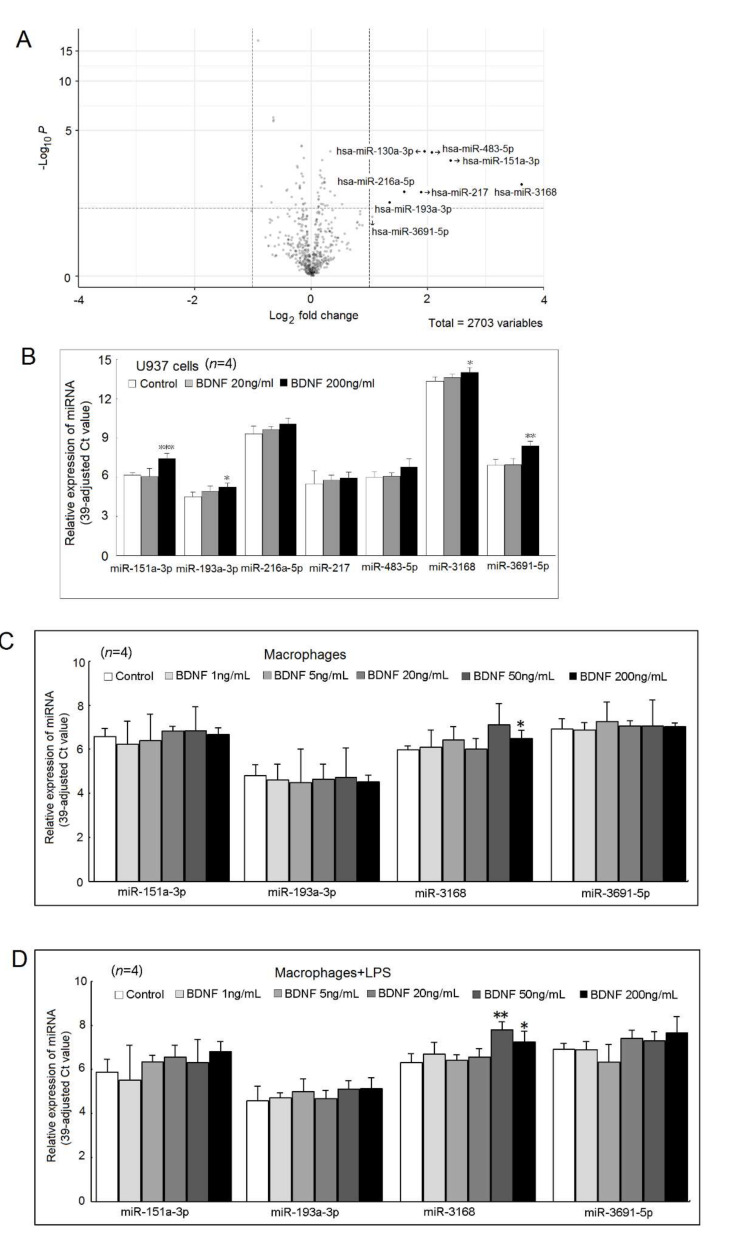
Differential expression of BDNF-regulated miRNA in U937cells cultured with BDNF (200 ng/mL) for 48 h or medium only. (**A**) Expression profiles of miRNAs in U937 cells cultured with BDNF (200 ng/mL) or medium only for 48 h, were evaluated using miRNA-Seq transcriptome analysis. Each scatter point represents the mean read count of miRNA in three repeats of each treatment; (**B**) Validation of potential candidates of BDNF-regulated miRNAs in U937 cells. Because the expression of miR-130a-3p was too low, we did not include it in the validation stage. Among the BDNF-regulated miRNAs, only miR-3168 was increased in (**C**) differentiated macrophages or (**D**) LPS-stimulated macrophages (* *p* < 0.05; ** *p* < 0.01; *** *p* < 0.001).

Using the next-generation sequencing (NGS) analysis, we found that several miRNAs were differently expressed after being cultured with a high concentration of BDNF (200 ng/mL) for 48 h (Figure 7A). The expression of miR-130a-3p was too low and therefore, we did not include it in the validation stage. After validation, we found that miR-151a-3p, miR-193a-3p, miR-3168, and miR-3691-5p were up-regulated by a high concentration of BDNF in U937 cells (Figure 7B). We found that the expression of miR-3168 was up-regulated in differentiated macrophages and LPS-stimulated macrophages in a high concentration of BDNF (Figure 7C,D) compared with the controls.

### 2.8. Identification of Protein Expression Regulated by miR-3168

**Figure 8:**
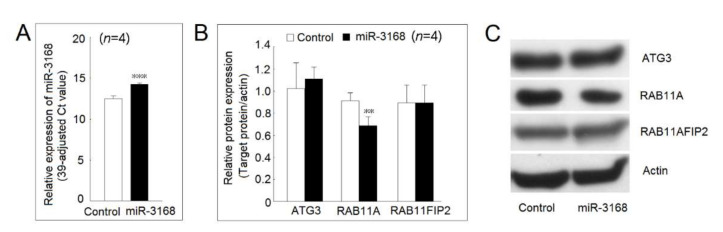
Identification of protein expression regulated by miR-3168. (**A**) The expression of miR-3168 was markedly increased in U937 after transfection with a miR-3168 mimic compared with those transfected with scrambled oligonucleotides as a control. (**B**) Autophagy-related 3 (ATG3), Ras-related protein Rab-11A (RAB11A), and RAB11 family interacting protein 2 (RAB11FIP2) were potential targets of miR-3168. After transfection of a miR-3168 mimic, the protein expression of RAB11A, but not ATG3 or RAB11FIP2 was decreased in U937 cells. (**C**) A representative example (** *p* < 0.01; *** *p* < 0.001).

After searching in the TargenScanHuman 7.2 (http://www.targetscan.org/vert_72/) (accessed on 14 November 2020) and miRDB (http://mirdb.org/) (accessed on 14 November 2020), we found that autophagy-related 3 (ATG3), Ras-related protein Rab-11A (RAB11A), and RAB11 family interacting protein 2 (RAB11FIP2) were potential targets of miR-3168. First, we demonstrated that the transfection of a miR-3168 mimic could cause a 3.4-fold increment in the miR-3168 expression level (Figure 8A). After transfection with a miR-3168 mimic, the protein expression of RAB11A, but not ATG3 or RAB11FIP2 was decreased in U937 cells (Figure 8B,C).

### 2.9. Functional Annotation of BDNF-Regulated miRNAs

**Figure 9:**
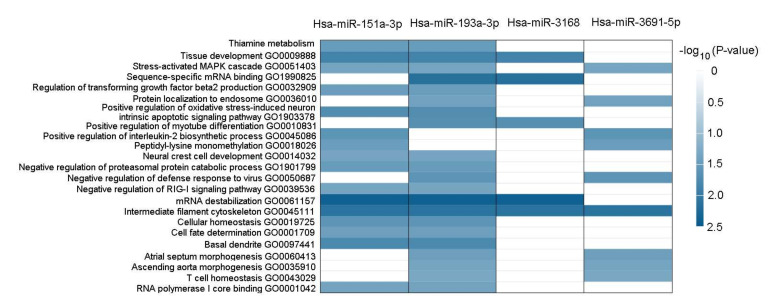
Pathways and gene ontology of the BDNF-regulated miRNAs analyzed by the miRNA enrichment analysis and annotation tool.

The possible pathway and gene ontology of the BDNF-regulated miRNAs, including mir-151a-3p, miR-193a-3p, miR-3168, and miR-3691-5p are shown in Figure 9. We found that the expression of miR-3168 was associated with the gene ontology of tissue development, sequence-specific mRNA binding, positive regulation of myotube differentiation, mRNA destabilization, and intermediate filament cytoskeleton.

## 3. Discussion

In our previous report, we found that serum BDNF was elevated in patients with rheumatoid arthritis and BDNF could promote *IL-2*, *IL-17*, and *IFN-γ* expression in Jurkat cells. However, in this study, we were surprised to observe that BDNF could suppress the TNF-α, IL-1β, and IL-6 secretion in macrophages and LPS-stimulated macrophages. Two previous reports showed that BDNF could promote M2 polarization of macrophages through repressing the STAT3 pathway and inhibit the expression of TNF-α and IL-1β [12,15] and our result was consistent with their findings. The most important point is that we confirmed that BDNF could suppress IL-1β and IL-6 secretion in human monocyte-derived macrophages.

In our p75NTR knockout study, we found that p75NTR was required for proinflammatory cytokines, including IL-1β, IL-6, and TNF-α secretion in macrophages. Therefore, the suppressive effect of BDNF on proinflammatory cytokines expression and secretion might be mediated through the other BDNF receptor, TrkB. Xu et al. demonstrated that BDNF could suppress the *TNF-α*, *IL-1β*, and *IL-6* expression via TrkB through MyD88/NF-κB and PI3K/AKT-signaling pathways in experimental pneumococcal meningitis model [16]. Liang et al. also showed that BDNF could suppress *TNF-α*, *IL-1β*, and *IL-6* expression through the TrkB/MAPK pathway in rats with spinal cord injury [17]. Our result is consistent with their reports. In addition, we noted that the effect of p75NTR blockade in the inflammatory response is still controversial. Düsedau et al. demonstrated that mononuclear cells in the brains with Toxoplasma infection in p75NTR double knockout mice increased the production of IL-10, IL-6, and IL-1α [18]. However, Lee et al. showed that blocking p75NTR reduced the pro-inflammatory response and inhibited TNF-α secretion of monocytes to LPS in vitro [19]. Our finding is consistent with their report. Different experimental conditions and different methods of inhibition of p75NTR might affect these results. The signaling pathway of BDNF and two receptors are highly complex. The p75NTR could bind not only to BDNF but also to other membrane neurotrophin families, including nerve growth factor (NGF), neurotrophin-3 (NT-3), and neurotrophin-4 (NT-4). The NGF-p75NTR axis has also been demonstrated to be involved in the inflammation response [20]. We showed that the knockout of p75NTR could markedly decrease TNF-α, IL-1β, and IL-6 secretion in macrophages and LPS-stimulated macrophages. It is possible that the knockout of p75NTR could also affect its binding to other neurotrophins, such as NGF, which is known to affect the IL-1β and IL-6 secretion in macrophages [21]. Recently, the blocking of NGF was demonstrated to be an effective therapy for treating osteoarthritis [22]. Currently, blocking p75NTR using a recombinant human protein has been developed for treating Alzheimer’s disease [23]. Both IL-6 and TNF-α play critical roles in the immunopathogenesis of rheumatoid arthritis. Our results suggested that blocking p75NTR could be another potential therapy for ameliorating inflammation in patients with rheumatoid arthritis.

We noted that the mRNA expression of *IL-1β* was slightly decreased in macrophages, and *IL-6* was slightly decreased in LPS-stimulated macrophages but had no effect on *TNF-α* mRNA expression after being cultured with BDNF. We found that miR-151a-3p, miR-193a-3p, miR-3168, and miR-3691-5p were up-regulated by a high concentration of BDNF (200 ng/mL) in U937 cells. Only the expression of miR-3168 was up-regulated in differentiated macrophages and LPS-stimulated macrophages by BDNF. We confirmed that miR-3168 could inhibit the protein expression of RAB11, which could mediate the transportation of cytokines [24,25]. This finding might explain the observation that BDNF suppressed the secretion of proinflammatory cytokines but had little effect on their mRNA expression.

There are three limitations to this study. First, the p75NTR knockout study was performed in U937 cells. Whether p75NTR is required for proinflammatory cytokines secretion in human macrophages remains to be determined. Second, a high concentration of BDNF is needed for the increased expression of miR-3168. Whether this phenomenon occurred under physiologic conditions remains to be elucidated. Third, the suppressive effect of low-dose BNDF (1 or 5 ng/mL) on the ERK phosphorylation is unexpected. Further studies are needed to clarify this observation.

## 4. Materials and Methods

### 4.1. Cell Culture

Differentiated macrophages were obtained from the oncogenic human monocyte cell line U937 (American Type Culture Collection, Manassas, VA, USA) by culturing with 500 ng/mL phorbol 12-myristate 13-acetate (Sigma-Aldrich, St. Louis, MO, USA) at 37 °C in a humidified atmosphere containing 5% CO_2_ for 48 h as previously described [26]. For analysis of mRNA expression, the differentiated macrophages were stimulated with 20 ng/mL lipopolysaccharides (LPS) (Sigma-Aldrich, St. Louis, MO, USA) at 37 °C in a humidified atmosphere containing 5% CO_2_ for 4 h. In addition, U937 cells, differentiated macrophages, and LPS-stimulated macrophages were cultured with culture medium only, different concentrations of BDNF (0, 1, 5, 20, 50, or 200 ng/mL) for 48 h, and the culture supernatants were then collected and stored at −80 °C for enzyme-linked immunosorbent assay (ELISA) analysis.

### 4.2. Preparation of Human Monocyte-Derived Macrophages

The study protocol was approved by the institutional review board of Dalin Tzu Chi Hospital, Buddhist Tzu Chi Medical Foundation (No. B10901029, 22 April 2020) and all participants signed informed consent. Heparinized venous blood from healthy individuals was mixed with one-fourth volume of 2% dextran solution (Sigma-Aldrich) and incubated at room temperature for 30 min. Leukocyte-enriched supernatants were layered over a Ficoll-Hypaque density gradient solution (specific gravity 1.077) (Pharmacia Biotech, Uppsala, Sweden). Peripheral blood mononuclear cells (PBMCs) were obtained from centrifugation. Then, monocytes were purified using the EasySep Human Monocyte Isolation Kit (Stemcell Technologies, Vancouver, Canada) and differentiated to macrophages using the M1-Macrophage Generation Media DXF (PromoCell, Heidelberg, Germany) according to the manufacture’s protocol [26].

### 4.3. Measurement of Gene Expression Levels by RT-PCR

Total RNA was extracted using the Quick-RNA MiniPrep Kit (Zymo Research, Irvine, CA, USA) according to the manufacturer’s manual. The concentration of RNA was quantified using the NanoDrop 1000 spectrophotometer (Thermo Fisher Scientific, USA). The mRNA expression levels of *IL-1β*, *TNF-α*, and *IL-6* were measured using a one-step RT-PCR kit (TaKaRa, Shiga, Japan) with an ABI Prism 7500 Fast Real-Time PCR system (Applied Biosystems, Waltham, MA, USA). Relative expression levels of mRNA were determined from the following equation: (39—threshold cycle [Ct] adjusted by the expression of 18S ribosomal RNA).

### 4.4. Enzyme-Linked Immunosorbent Assay (ELISA)

The concentration of TNF-α, IL-1β, and IL-6 in the culture supernatants was measured using an ELISA kit (Biosensis, Adelaide, Australia) according to the kit protocol.

### 4.5. Cell Viability and Proliferation Using the Mitochondrial Dehydrogenase Cleavage Assay

Cells were cocultured with low or high concentrations of BDNF for 48 h, then 10 μL WST-1 (Roche Applied Science, Basel, Switzerland) was added and incubated for 60 min. The intensity of color formation was detected using Anthos Zenyth 3100 multimode fluorometer (Anthos Labtec Instruments GmbH, Salzburg, Austria).

### 4.6. Preparation of Surface Membrane Extract

Surface membrane proteins from cells were extracted using the ProteoExtract native membrane protein extraction kit (Calbiochem, San Diego, CA, USA). The concentration of the extracted membrane proteins was measured by the Bradford method. The expression of TrkB and p75NTR was detected via Western blotting using mouse monoclonal antibodies against human TrkB or p75NTR (BioLegend, San Diego, CA, USA). The expression of transferrin receptor (TfR) was used as an internal control using mouse monoclonal antibody against transferrin receptor (Abcam, Cambridge, UK).

### 4.7. Western Blot Analysis

Western blot analysis was performed as previously described [27]. Briefly, cell lysates were electrophoresed and transferred to a polyvinylidene difluoride sheet (Sigma-Aldrich). The membranes were blocked with 1% skim milk solution and incubated with primary antibodies followed by respective HRP-conjugated secondary antibodies. The blots were detected using chemiluminescence (GE Healthcare, Little Chalfont, UK). Densitometric analysis of band intensities was performed using Image J software (version 1.42; http://rsb.info.nih.gov/ij). The antibodies used for Western blot analysis included rabbit monoclonal antibodies against ERK1/2, phospho-ERK1/2 (Thr202/Tyr204), p38, phospho-p38 (Thr180/Tyr182), JNK, phospho-JNK (Thr183/Tyr185), c-Jun, phospho-c-Jun (Ser63), and goat-anti-rabbit IgG conjugated with horseradish peroxidase (Cell Signaling Technology, Danvers, MA, USA). Anti-β-actin antibody was used as an internal control (Sigma-Aldrich).

### 4.8. NGS for miRNAs

The expression profile of miRNAs in U937 cells cocultured with BDNF (200 ng/mL) for 48 h or culture medium was identified and compared using NGS analysis. Total RNA was extracted using Trizol^®^ Reagent (Invitrogen, USA) according to the manufacturer’s manual. RNA purified was quantified at OD_260nm_ using the ND-1000 spectrophotometer (Nanodrop Technology, USA), and the quality was evaluated using the Bioanalyzer 2100 (Agilent Technology, USA) with the RNA 6000 LabChip Kit (Agilent Technologies, USA). The sample libraries were prepared using the QIAseq miRNA Library Kit (QIAGEN, Hilden Düsseldorf, Germany) according to the manufacturer’s protocol. Adaptors are ligated sequentially to the 3′ and 5′ ends of miRNAs. Universal cDNA synthesis with unique molecular identifier tagging, cDNA cleanup, library amplification, and library cleanup was performed. Libraries were sequenced on an Illumina instrument (75 cycles, single-end read). The Illumina BCL2FASTQ conversion software v2.20 was used to process the sequencing data. Trimmomatic was used to clip the 3′ adaptor sequence, trim, or remove the reads according to quality scores. Trimmed reads shorter than 18 nucleotides were discarded [28]. Qualified reads after filtering low-quality data were analyzed using miRDeep2 software [29] for aligning reads to the reference genome downloaded from the University of California Santa Cruz (UCSC) Genome Browser Database. Only reads that mapped perfectly to the genome ≤ 5 times were used for miRNA detection because miRNAs usually map too few genomic locations. The miRDeep2 software was used to estimate the expression levels of known miRNAs and to identify novel miRNAs. The small RNA library construction, NGS deep sequencing, and bioinformatic analysis were carried out by Welgene Biotech. Co., Ltd. (Taipei, Taiwan). The functional analysis of BDNF-regulated miRNAs was performed by the miRNA enrichment analysis and annotation tool (https://ccb-compute2.cs.uni-saarland.de/mieaa2) (accessed on 6 October 2021) [30].

### 4.9. Measurement of miRNAs Expression

The expression levels of miRNAs were quantified using a real-time PCR-based method following a protocol described previously [31]. The U6 small nuclear RNA was used as a normalization control.

### 4.10. Preparation of p75NTR Knockout U937 Cells

U937 cells were electroporated with control CRISPR-Cas9 plasmids or CRISPR-Cas9 plasmids containing gRNA that targeted p75NTR (Santa Cruz Biotechnology). The Gene Pulser MXcell electroporation system (Bio-Rad Laboratories, Hercules, CA, USA) was used at 210 V and 960 μF. The cells were then cultured in Iscove’s Modified Dulbecco’s Medium (IMDM) (Invitrogen, Carlsbad, CA, USA) with 10% fetal bovine serum (Invitrogen) and 0.3 μg/mL puromycin (Sigma-Aldrich, St. Louis, MO, USA). Cells that survived puromycin selection were isolated and validated by Western blot analysis.

### 4.11. Statistical Analysis

Summary data were expressed as the mean and standard deviation. Student’s *t*-test or non-parametric Wilcoxon signed-rank test was performed using Stata/SE version 8.0 for Windows (StataCorp, College Station, TX, USA). Two-tailed *p* values < 0.05 were considered significant.

## 5. Conclusions

In conclusion, we found that BDNF could suppress the secretion of proinflammatory cytokines in macrophages and inhibit JNK and c-Jun phosphorylation. The knockout of one of the BDNF’s receptors, p75NTR, could effectively suppress the proinflammatory cytokine secretion in macrophages. BDNF could upregulate the expression of miR-3168 with Ras-related protein Rab-11A as its target. We proposed that targeting p75NTR could be a potential therapeutic strategy for treating inflammatory conditions.

## Data Availability

The datasets analyzed during the current study are available from the corresponding author on reasonable request.
